# Cellular Players in Skeletal Muscle Regeneration

**DOI:** 10.1155/2014/957014

**Published:** 2014-03-23

**Authors:** Laura Cristina Ceafalan, Bogdan Ovidiu Popescu, Mihail Eugen Hinescu

**Affiliations:** ^1^Department of Cell Biology and Histology, School of Medicine, “Carol Davila” University of Medicine and Pharmacy, 8 Eroii Sanitari, 050474 Bucharest, Romania; ^2^Department of Molecular Medicine and Neuroscience, “Victor Babes,” Institute of Pathology, 99-101 Splaiul Independentei, 050096 Bucharest, Romania; ^3^Department of Neurology, Colentina Clinical Hospital (CDPC), School of Medicine, “Carol Davila” University of Medicine and Pharmacy, 19-21 Sos. Stefan cel Mare, 020125 Bucharest, Romania

## Abstract

Skeletal muscle, a tissue endowed with remarkable endogenous regeneration potential, is still under focused experimental investigation mainly due to treatment potential for muscle trauma and muscular dystrophies. Resident satellite cells with stem cell features were enthusiastically described quite a long time ago, but activation of these cells is not yet controlled by any medical interventions. However, after thorough reports of their existence, survival, activation, and differentiation there are still many questions to be answered regarding the intimate mechanism of tissue regeneration. This review delivers an up-to-date inventory of the main known key players in skeletal muscle repair, revealed by various models of tissue injuries in mechanical trauma, toxic lesions, and muscular dystrophy. A better understanding of the spatial and temporal relationships between various cell populations, with different physical or paracrine interactions and phenotype changes induced by local or systemic signalling, might lead to a more efficient approach for future therapies.

## 1. Introduction

Adult mammalian skeletal muscle is a dynamic tissue in terms of remodelling, repair, and regeneration. The cells may undergo physiological changes based on everyday physical activity (atrophy, hypertrophy, or fibre type switch). Adult skeletal muscle cells are also able to repair focal damages induced by muscle contraction to the sarcolemma or myofibrils, with no inflammatory reaction and preservation of the histological features.

Moreover, due to the superficial location, skeletal muscle tissue is constantly subjected to different grades of traumatic injuries that may cause necrosis of entire cells or only of fibre segments. New myofibres will be formed in the process of* muscle regeneration*.

Skeletal muscle regeneration is a complex phenomenon that involves many regulatory processes that require a close collaboration of two major cellular categories: stem/progenitor cells and surrounding supporting interstitial cells. By direct contact or by releasing soluble factors, different types of interstitial cells are responsible either for the maintenance of the stem cell niche in the normal tissue or for recruiting of different pools of stem/progenitor cells during muscle regeneration.

**Figure 1:**
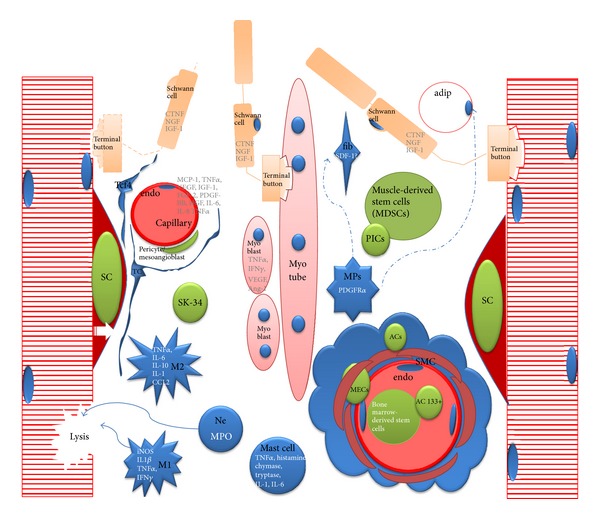
Synoptic view on the skeletal muscle interstitial space. Different stem cell populations become activated after acute injury (green), differentiate, and fuse to form myotubes with the support provided by various interstitial and blood derived cells (blue) either by physical contact or paracrine signalling. Satellite cell (*SC*); endothelial cell (*endo*); adipocyte (*adip*); mesenchymal progenitors (*MPs*) include the PDGFR*α*+ progenitors, the FAPs, and Tie2+; skeletogenic progenitors; PW1+/Pax7–interstitial cells (*PICs*); SK-34 cells (*SK-34*); telocytes (*TCs*); SDF-1 skeletal muscle-derived fibroblast (*fib SDF-1*); macrophages (*M1/M2*); neutrophils (*Ne*); myogenic endothelial cells (*MECs*); Tcf4 positive fibroblasts (*Tcf4*); adventitial cells (*ACs*); smooth muscle cells (*SMC*).

This review focuses on recent advances in the cellular and molecular biology of skeletal muscle regeneration based on cell populations described to play a role in this process. This “social” context is summarized in Table 1 and Figure 1.

## 2. Steps in Skeletal Muscle Regeneration following Acute Injuries

Mechanical acute injuries lead to muscle fiber destruction by disruption of plasma membrane and basal lamina, subsequent calcium inflow, and necrosis by autodigestion or eventually apoptosis.

Animal studies provided evidence that the healing process after direct trauma requires* three steps* following* necrosis*, interrelated and time dependent, as described below [1, 2].

### 2.1. Degeneration and Inflammatory Response

It starts within the first minutes following injury and lasts for up to 2 weeks. The affected site is invaded by leukocytes and macrophages, actively secreting cytokines and growth factors that not only amplify the inflammatory response, but also take part in the second phase of muscle regeneration.

### 2.2. Regeneration/Repair Phase

It initiates in the first week after injury and peaks at 2 weeks, and consists of three major stages starting with the* activation* and* differentiation* of muscle* stem cells* followed by maturation of the myofibres and paralleled by formation of new vessels by* angiogenesis* to revascularize the newly formed myofibres. Those key processes are orchestrated by a large panel of signals originating in the blood stream or in the local cellular environment.

### 2.3. Scar Formation

It begins during the 2nd week after injury and increases over time. The appearance of scar tissue impairs complete muscle regeneration.

Naturally, this time line can vary greatly depending on species and within the same species depending on injury type and severity or even on the individual metabolic state.

## 3. Muscle Stem/Progenitor Cells

### 3.1. Satellite Cells

The most studied and commonly accepted progenitor cell population in postnatal skeletal muscle is still represented, even after 50 years since their discovery, by the satellite cells [3]. Such cells were originally identified by electron microscopy based on their particular location, accompanying adult skeletal muscle fibres, unsheathed by their basal lamina. It was estimated that such cells account for 2–5% of identifiable nuclei [4] located under the basal lamina in adult muscle [5].

Satellite cells are responsible for the early growth of the myofibre and then they become mitotically quiescent [4]. Throughout adult life they are frequently recruited either for fibre maintenance or, when needed, for cell hypertrophy and focal repair through proliferation and fusion with the myofibre [6]. During adult muscle regeneration they differentiate to myogenic precursor cells (MPCs) which will divide repeatedly before fusing into myotubes.

Early histological studies estimated that the proportion of satellite cells drops from 30–35% in the postnatal life to 1–4% in the adult life in mice [6]. Following studies suggested that in growing muscle there are two subpopulations of satellite cells: a fast-dividing subpopulation, responsible for fibre growth and a slow-dividing one that could function as the source of the former or could be formed by different cells. The overall satellite cell number decrease over time could be explained by the waste of the fast-dividing subset as they change from asymmetric to symmetric division, so that most adult satellite cells will derive from the slow-dividing population. However, in normal adult muscle this population will remain constant even after recurrent cycles of necrosis-regeneration, which clearly suggests that the satellite cell pool is maintained by self-renewal.

At first, satellite cells were considered as muscle precursor cells derived from a population of circulating bone marrow [7] or resident stem cells [8]. Previous studies using either bone marrow-derived cells or dissociated satellite cells did not show a significant contribution to the satellite cell compartment in animal models of muscle-induced injury and they required a large number of transplanted cells [7].

The mesenchymal multipotent stem cell nature of satellite cells was also suggested by further studies based on their osteogenic and adipogenic differentiation potential, besides the well-known myogenic one [9]. Recently, this theory started to be questioned as other mesenchymal progenitors, expressing PDGFR*α* and located in the interstitium, represent the only cell population in the adult skeletal muscle capable of differentiation along adipogenic [10] or osteogenic lineage [11].

Though, stem cell core features like proliferation, self-renewal, and differentiation capacity were eventually demonstrated over the years for the satellite cells through various* in vitro* or* in vivo* studies [12]. One of the most convincing evidences in this respect was based on* in vivo* transplantation of single fibres where no more than seven satellite cells regenerated and repopulated radiation-ablated muscles of dystrophic* mdx*-nude mice [13]. However, differences have been noted regarding the behaviour of satellite cells depending on the donor muscle group, which were suggested to result from local environmental factors.

Such studies brought into light two very important aspects regarding satellite cells and their proper function in terms of activation and recruitment during tissue regeneration: the heterogeneity of this population and the importance of the stem cell niche.

#### 3.1.1. Phenotype

Many recent studies focusing on the identification and prospective isolation of satellite cells reported the expression of various markers on satellite cells [14]. Among them, paired box transcription factor Pax7 is the only marker specifically expressed on both quiescent and activated satellite cells. Previous studies on* Pax7-null* mice proved that the muscle develops, but the postnatal growth is compromised; thus, Pax7 appears to be essential for satellite cell formation [15]. Unexpected evidence came from a recent study demonstrating that when Pax7 is inactivated in adulthood, the satellite cells can still support muscle regeneration [16]. Apparently, Pax7 is required in the perinatal life only until satellite cells become quiescent. This study points out an interesting facet of any adult stem cell system; that is, the genetic requirement changes with age, so basically stem cells also do have an age. However, if Pax7 expression is required in skeletal muscle acute injury, regeneration is still a matter of debate [17]. Very recent data on conditional knock-out mice conclude that Pax7 is a prerequisite during regenerative myogenesis [18] for satellite cells proliferation and differentiation regardless of age [19].

Most of the other markers, presented in Table 2, are also expressed on other cell types present in the adult skeletal muscle and therefore their presence should be correlated with the specific location of such cells, under the basal lamina of skeletal muscle fibres. Before activation, quiescent satellite cells do not express muscle-specific proteins, like the members of the myogenic-regulatory-factor family, but the phenotype changes upon activation.

Recently, by developing a new monoclonal antibody, SM/C-2.6, Fukada et al. [36] identified quiescent M-cadherin positive satellite cells in their location and further enriched them from adult mouse muscle. Functional studies were also performed and proved that this antibody could be useful as a powerful tool for future investigations.

#### 3.1.2. Heterogeneity

Proofs for the diversity of the myogenic compartment have been provided by both* in vitro* and* in vivo* models [6, 110].

*In vitro* approaches based on single muscle fibre culture in suspension proved that satellite cells proliferate at different rates and define high (HPC) and low (LPC) proliferative rate clones, with fixed ratio at single fibre level [110]. The study suggested that HPC represent a source of adipogenic tissue within the skeletal muscle in pathological conditions as they spontaneously differentiated to adipocytes, but they can be conditioned towards a massive myogenic differentiation if cocultured with LPC. The existence of such a paracrine effect might explain why such a spontaneous adipogenic trigger of differentiation is not present in healthy muscles.

More recent studies suggested that all those differences are orchestrated by the special environment provided by the niche. The niche is a dynamic microenvironment; it not only maintains stem-cell quiescence, but also manages the activation of stem cells when required.

Apparently, the orientation of the division plane within the niche is responsible for maintaining this diversity; planar division generates two identical daughter cells, while apical-basal division generates asymmetric cell fates.

The intrinsic heterogeneity was demonstrated by immunohistological analysis of phenotypic expression, especially based on myogenic regulatory factors (MRFs). Thus, the basal cell expresses only Pax7, maintains a stem cell identity, and functions as a stem cell reservoir, whereas the cell that loses contact with the basal lamina commits to a myogenic fate and has a different profile, expressing both Pax7 and Myf5 [111]. Pax7+/Myf5+ cells can undergo limited symmetric proliferation to generate myonuclei. This process is carefully regulated by Notch signalling pathway in order to maintain the self-renewal property of satellite stem cells under normal conditions. Previous studies showed that Notch-1 promotes the proliferation of satellite cells (Pax3+/Desmin−/Myf5−/MyoD−), whereas its inhibition by Numb, which prevents Notch translocation into the nucleus, leads to the commitment of the progenitor cells to the myogenic cell fate (Pax3−/Desmin+/Myf5+) and to their myogenic differentiation [39].

More recently, other satellite cell subpopulations have been defined based on variation in the expression of nonspecific myogenic markers such as nestin [109], CXCR-4 and *β*1-integrin [40], or ABCG2 and Syndecan-4 [108].

Poor survival and engraftment upon satellite cells transplantation could also suggest that this heterogeneous population could contain only a small proportion of cells with real stem cell features [112].

#### 3.1.3. Activation Milestones

Satellite cell activation and differentiation during regeneration recapitulate embryonic developmental steps based on similar regulatory mechanisms, but in a completely different environment.

The* activation* of satellite cells and their subsequent* differentiation* along the myogenic lineage are controlled by various myogenic regulatory factors (MRFs): Myf5, MyoD, myogenin, and MRF4. The activation of surviving satellite cells takes place during muscle fibre degeneration. By asymmetric division, committed satellite cells already express Myf5 [111]. Shortly after activation, MyoD is rapidly induced* in vivo* in satellite cells that are selected for differentiation and the cells migrate out of the sublaminar niche. The proliferating cells known as MPCs or myoblasts downregulate Pax7 and commit to myogenic differentiation, expressing myogenin [100] and MRF4 [99, 102]. They undergo multiple rounds of mitosis before* terminal differentiation*. During terminal differentiation, myoblasts withdraw from the cell cycle, elongate, express muscle-specific genes at high levels (*α*-actin, myosin heavy chain), and fuse to multinucleated muscle cells to form the mature muscle fibre [101].

Regarding the activation trigger, there are still many signals that could be taken into account.

Intrinsic signals could include the production of sphingosine-1-phosphate from the inner leaflet of the plasma membrane [113]. However, its synthesis is mandatorily needed for entering the cell cycle. Extrinsic signals could be either mechanical, which, in turn, can trigger synthesis of nitric oxide that leads to hepatocyte growth factor and follistatin release or other promyogenic growth factors and cytokines involved in satellite cell activation [102].

### 3.2. Other Myogenic Cell Populations

The satellite cell is still in focus for cellular therapies in various muscle diseases but the clinical trials based on satellite cell transplantation showed poor survival, migration, and insufficient self-renewal. Therefore alternative approaches are required.

Studies focusing on transplanting stem cells with different origins showed that they also participate in muscle regeneration upon engraftment, at least to some extent. The logical reasoning question was whether satellite cells are the only stem cell source during muscle regeneration.

#### 3.2.1. Nonresident (Circulating) Stem Cells

First evidence that muscle regeneration could involve nonresident stem cells came from transplantation studies with* bone marrow-derived stem cells (BMDC)* into lethally irradiated mice [114]. After transplantation, bone marrow stem cells migrated toward injured area and differentiated into myogenic progenitors that regenerated damaged fibres.

Similar experiments were carried out on animal models of Duchenne's muscular dystrophy after fluorescence-activated cell sorting (FACS) purification of both Hoechst 33342-stained haematopoietic stem cells and mononuclear cells isolated from skeletal muscle tissue. The subpopulation that excluded Hoechst was named* side population*. Hematopoietic side population (HSP) cells were found positive for hematopoietic stem cell markers Sca-1, CD43, c-kit, and CD45 [42].

After intravenous injection, bone marrow SP cells reconstructed the hematopoietic compartment of lethally irradiated mice and engrafted in skeletal muscle but provided a low dystrophin expression post-transplantation, insufficient for clinical recovery.

Further studies, using BMDC expressing GFP, showed that upon transplantation into irradiated mice GFP positive cells could be detected in the satellite cell niche, where they became satellite cells since they expressed muscle specific proteins and self-renewed* in vitro* [7]. Using a model of exercise-induced damage, these studies demonstrated that such cells were incorporated into muscle fibres at a higher rate than previously reported.

Another subpopulation of circulating stem cells expressing the cell surface antigen CD133 also expressed myogenic markers. In this line of evidence,* CD133+ cells* can not only repopulate bone marrow and differentiate into endothelial cells upon transplantation, but they can also undergo* in vivo* myogenesis after cocultivation with myoblasts and injection into dystrophic muscles [63]. Such studies demonstrated that this cell type participates in muscle regeneration and even repopulates satellite cell niche.

#### 3.2.2. Myogenic Cells That Reside in the Skeletal Muscle Interstitium

*Interstitial muscle-derived stem cells* (MDSCs) have been identified in the last few years after being isolated* in vitro* by various modern methods. For some of those stem cell populations,* in situ* studies highlighted their localizations in skeletal muscle interstitial space.

Studies by Lepper et al. [17] used conditional gene inactivation and showed that Pax7+ cells are the only effective source of regenerative myonuclei after acute injury. Therefore, even though many other progenitor cell populations might have the potential to interfere with the muscle regeneration process, it seems that Pax7+ cells are the regeneration effectors. One cannot exclude a phenotype versatility of all these progenitor populations upon specific local stimuli.

One population of interstitial stem cells, which has been shown to home selectively and preferentially to skeletal muscle after injury, is represented by* mesoangioblasts*, a subset of vessel-associated stem cells that differentiate into several mesenchymal cell types, skeletal muscle included [47, 50].

In contrast to other types of circulating stem cells, hematopoietic stem cells included, such cells naturally migrate to dystrophic muscle. After systemic delivery into the areas of muscle injury and inflammation, they have been shown to migrate outside the vessel and to restore the structure and function in a mouse model of muscular dystrophy [47] and in a dog model with alterations of dystrophin gene that develops the full range of human pathology [48].

Several studies demonstrated that their migratory capacity can be increased by reconditioning with soluble molecules such as HMGB-1 [115], TNF-*α* (through MMP- and CD44-dependent mechanism), or SDF-1 (through MMPs and *α*v integrins) [47].

Recent studies showed that mesoangioblasts are recruited by infiltrating polarized macrophages that at first, during the inflammatory phase, secrete, among other soluble factors, HMGB1 and TNF-*α* (M1 cells) which favour in turn the homing of circulating progenitors and later on, during the resolution phase, support this action by MMP-9 secretion (M2 cells) [49]. Another study showed that macrophages regulate differentiation of mesoangioblasts through IL-10/IL-10R signalling, both* in vitro* and* in vivo* [116].

Another fraction of the MDSCs is represented by* muscle side population (MSP) cells*. MSP cells were also isolated by FACS, based on Hoechst exclusion and Sca-1 expression directly from skeletal muscle tissue. Immunolabelling assays showed that they do not express c-kit, CD43, and CD45 as the HSP or any satellite cell markers like CD34 [42]. The molecular determinant of the SP phenotype is Abcg2, a member of the ABC transporter family [56].

Based on Sca-1 expression and later on based on Abcg2 expression in the interstitium, MSP cells were located outside the basal lamina of the muscle fibre, associated with endothelium and outer layer of blood vessels [8, 56].

Previous studies showed that these cells could improve dystrophin expression in mdx mice and reconstitute the haematopoietic compartment after intravenous injection into irradiated mice [117].* In vitro*, such cells underwent hematopoietic differentiation but did not differentiate into myocytes. After intramuscular injection, they seem to be able to give rise to myocytes and satellite cells only [8].

More recent studies, using Abcg2 null mice, proved delayed immune response and muscle regeneration concomitantly with a decreased number of Pax7+ satellite cells. However, lineage tracing experiments showed that Abcg2 labelled cells give rise mainly to vessel-associated cells (endothelial cells and pericytes) and have a limited myogenic activity upon injury. Based on phenotypic evaluation, the authors conclude that Abcg2 positive cell population is heterogeneous, including MSP cells together with other vascular-interstitial cells and also circulating progenitor cells, which home to skeletal muscle upon injury [56].

*PW1/PICs*. Another population of muscle-derived stem cells is PW1+/Pax7− interstitial cells (PICs). They were first detected in the interstitium of skeletal muscle tissue while studying the effect of TNF on muscle homeostasis and stem cells. Such cells express PW1, a cell stress mediator implicated in TNF-NF*κ*B signalling and p53-mediated cell stress pathways, along with Sca-1 and CD34. Apparently, the overall effect of TNF*α* administration is a delay in skeletal muscle regeneration and this population was the one to respond to TNF*α* by caspase activation. Moreover, the regeneration impairment could be overcome by caspase inhibition suggesting at least a regulatory role for these cells [43].

New data proved that such cells do not express other muscle satellite cells markers such as Pax7 or MyoD and based on lineage tracing experiments they are not derived from satellite cell lineage.* In vitro* testing showed that they are bipotential progenitors giving rise to both smooth and skeletal muscle cells. Moreover, they also presented a high self-renewal capacity, as another stem cell feature.

Functional studies demonstrated that they are also myogenic* in vivo*, participating in tissue regeneration.

Studies on constitutive Pax7 mutant mice showed a decrease in satellite cells, as Pax7 is required for satellite cell formation, but with a proportional increase in PICs during postnatal growth.* In vitro* testing demonstrated that, in such cases, PICs could only differentiate into smooth muscle cells, suggesting the requirement of Pax7 for their enrolment into skeletal muscle lineage [37].

Recently, a study of Pannerec et al. [44] demonstrated that PICs share the mesenchymal stem cell profile and can also differentiate into adipose cells. Such data open the discussion on to what extent this population overlaps with other populations that have been newly described in the skeletal muscle interstitium, such as, for instance, the interstitial adipogenic progenitors.

*SK-34 cells* are another population of MDSCs isolated by FACS from mouse skeletal muscle tissue. They are* CD34* positive cells located in the interstitium, outside the basal lamina, which do not express myogenic markers (MyoD, myf-5, myf-6, myogenin, M-cadherin, Pax-3, or Pax-7) or CD45. Most of them were positive for Sca-1 and negative for other endothelial markers (CD14, CD31, CD49, CD144, and Flk1) and showed multilineage potential (myogenic, endothelial, and adipogenic) [46].

CD34 positive cells isolated from GFP transgenic mice were transplanted into tibialis anterior (TA) muscles of male immune-deficient NOD/SCID mice. The results showed that they are able to differentiate into myocytes, vascular, and Schwann cells* in vivo*, contributing to the coordinated reconstitution of muscle fibres, blood vessels (pericytes, smooth muscle cells, and endothelial cells), and peripheral nerves, with significant structural and functional recovery after transplantation [118].

*SASCs*—*slow*-*adhering stem cells*—were isolated* in vitro* based on a special preplating technique from the injured muscle. This heterogeneous population showed an increased proportion of Sca-1 and CD34 positive cells, an increased migration, proliferation, and differentiation potential, and better engraftment in mdx/scid mice. Such cultures also showed an upregulation of multiple genes responsible for multipotency, development, and muscle regeneration like Notch1, STAT3, Msx1, Pax3, and MMP2 [45].

#### 3.2.3. Vascular Progenitor Cells

One of the newest emerging concepts in stem cell biology is that blood vessels represent a systemic source of progenitor cells [52, 119]. Multipotent adult stem cells have been isolated from all layers of blood vessel wall in skeletal muscle and various other organs: intima contains endothelial progenitor cells (MECs), media of small blood vessels contains pericytes, and in large vessels smooth muscle cell progenitors (SMPCs), and the outermost layer contains adventitial cells (ACs) [57, 61, 120]. The intersection of these two research areas, vascular progenitors and skeletal muscle biology, opens new and exciting perspectives for skeletal muscle regeneration.

*(1) Myogenic Endothelial Cells (MECs)*. MECs have been recently identified* in situ* by confocal microscopy based on coexpression of satellite cells markers (Pax7 and CD56) and endothelial markers (von Willebrand factor, VE-cadherin (CD144), UEA-1 receptor, and CD34) in between skeletal muscle fibres from human muscle biopsies, where blood vessels reside.

Subsequently, CD56+CD34+CD144+CD45− cells have been isolated by FACS as a scant cell population. They proliferated and survived in long-term cultures and were not tumorigenic. Upon transplantation, they were able to regenerate muscle fibres in SCID mice skeletal muscles after cardiotoxin-induced injury more effectively than skeletal myoblasts. They could be clonally cultured and showed myogenic osteogenic and chondrogenic differentiation potential [51, 52].

*(2) Pericytes*. Pericytes are the mural cells of the smallest division of the vascular system, the microvessels. They were regarded for a long time as structural elements, providing stability for these vessels and also being endowed with contractile properties. Not only are they enclosed in the endothelial cells basal lamina, but they even establish close contacts with endothelial cells. The interplay between endothelial and pericytes has recently come into focus as a central process in the regulation of vascular formation, stabilization, and maturation as long as disputed processes such as tissue remodelling and repair rely on angiogenesis.

Nowadays, pericytes emerge as a heterogeneous population in terms of origin, morphology, and marker expression [53].

In addition to their ability to modulate their phenotype along the pericyte-smooth muscle cell axis during vessel growth and remodelling, pericytes have greater phenotypic plasticity, being viewed as multipotent progenitor cells with capacity to differentiate into adipocytes, osteoblasts, and chondrocytes [121].

Several studies reported their involvement in repair processes in various injured tissues as they become activated and differentiated into adipocytes, chondrocytes, Leydig cells, and even myoblasts [55, 122].

This process can be regarded as differentiation, as pericytes have stem cell features, or transdifferentiation of a differentiated cell toward a different phenotype, and this is clearly a matter of debate. However, a hypothetical continuum, from mesenchymal cells to extracellular matrix secreting fibroblasts and to blood vessel contractile phenotypes (pericytes or smooth muscle cells), could be taken into consideration.

Perivascular CD146+ pericytes isolated from skeletal muscle and nonmuscle tissues showed mesenchymal stem cells features with long-term myogenic potential both* in vitro* and* in vivo*, after transplantation into SCID-mdx or SCID-NOD cardiotoxin injured mice [57]. Lineage tracing experiments with tissue nonspecific alkaline phosphatase CreERT2 mice proved that alkaline phosphatase positive vessel-associated cells-pericytes contribute to postnatal muscle growth and satellite cells pool and their contribution is highly increased during tissue regeneration [58].

## 4. Cells of the Stem Cell Niches

Most data on potential stem/progenitor cells populations involved in skeletal muscle regeneration come from* in vitro* studies after isolation, characterization, and transplantation. Less is known about their distribution and the microenvironment needed for their maintenance, activation, and differentiation upon injury. Up to now, there are three potential stem cell niches that could be taken into consideration in skeletal muscle tissue: the satellite cell niche, specific for skeletal muscle, and two vascular stem cell niches (“universal” stem cell niches), one in the subendothelial zone and the second in the adventitial vasculogenic zone [119].

Muscle satellite cells niche is a polarized microenvironment structured by the basal lamina unsheathing the muscle fibre. The basal lamina anchors the basal side of satellite cells through laminin—*α*7*β*1 integrin receptor [111]. The apical pole of satellite cells is adjacent to the myofibre, where it is anchored by cell adhesion molecule M-cadherin. Integrity and composition of this niche impact on the repair process by providing the appropriate migration substrate and signals for satellite cells. Direct contact along with soluble factors released by neighbouring nonmuscle cells represents signals that are conferred to satellite cells [33, 123]. Most likely, direct contact with the myofibre is necessary in order to maintain them in a quiescence state, with low requirement for growth factors. The maturation compartment involves different types of immune and stromal cells that will provide both cell-to-cell contact and soluble factors required for satellite cell activation, proliferation, and differentiation. Additional support is provided by the particularly rich capillary network. There is a close association between satellite cells and blood vessels, with 88% of the satellite cells at less than 21 *μ*m away from a capillary [29, 124]. Besides being a nutrient supply, some studies suggest that there is a crosstalk between activated satellite cells and endothelial cells during differentiation that supports angiomyogenesis, most probably through soluble factors secretion [33]. Endothelial cells will provide insulin-like growth factor (IGF)-1, fibroblast growth factor (FGF), hepatocyte growth factor (HGF), and vascular endothelial growth factor (VEGF). In turn, differentiating myoblasts promote angiogenesis.

Another important regulation seems to be dependent on the neuromuscular junction [35] and periendothelial cells. Periendothelial cells promote the return to quiescence of a subset of satellite/myogenic cells and maintain their quiescence through Angiopoietin-1/Tie-2 signalling [125].

A new line of evidence suggested that in skeletal muscle the perivascular compartment represents a complex microenvironment, with more elaborated functions. A new concept emerged; that is, the subendothelial zone in small vessels and especially the area between media and adventitia and tunica adventitia itself in large vessels provide niche-like environments for resident progenitor cells involved in growth, remodelling, and repair of the blood vessel wall [119, 126].

*Adventitial Cells (ACs)*. So far, the adventitia was considered only as an assembly of fibroblasts, nerves, and microvessels travelling through an extracellular matrix. Recent studies proved that progenitor cells with the ability to form vascular structures are present in both murine and human blood vessel adventitia and in both arterial and venous blood vessel from various locations. In arteries wall, they were detected at the border zone between media and adventitia in the so called “vasculogenic zone” based on marker profile. CD34+/CD31− cells represent a source of endothelial progenitor cells (VW-EPCs) that could form capillary sprouts expressing VEGFR2 (KDR), Tie2, VE-cadherin, occludin, and CEACAM1 as they become engaged on the endothelial cell lineage. Moreover, this zone contains other cell subpopulations (CD45+ mononuclear cells and CD68+ macrophages) suggesting that most probably they all derive from a resident, scarce adult multipotent cell population [64].

Other studies proved that adventitial layer contains clusters of CD34+, Sca1+, c-kit-, Flk1+, and CD140b+ cells. They seem to have a differentiation potential toward endothelial cells, mural cells, adipocytes, and osteogenic cells [62, 127].

CD34+/CD31− progenitors were also identified in human saphenous vein adventitia, around vasa vasorum. Such cells coexpressed pericytes markers such as NG2 and PDGFR*β*. These saphenous vein-derived progenitor cells (SVPs) were further sorted by FACS, propagated, and characterized* in vitro*, proving clonogenic and multilineage potential [120].

It is still not clear yet if the same progenitors act as both vascular and tissue-specific progenitors nor if different cell populations settle in large, individual vessels or intraorgan vessels.

However, the adventitia environment should be also taken into account not only for the angiogenic/vasculogenic potential but also for analysing the regeneration process in skeletal muscle, as long as there is clear evidence that some vessel-associated cells could participate in this complex process.

## 5. Signalling Cells for Myogenic Stem Cell Activation/Differentiation and Angiogenesis

*Stem cell activation* and* angiogenesis* are central, complex, and coordinated processes for muscle regeneration, far from being reasonably elucidated.

Besides searching for the most appropriate type of stem cell for therapy, there is a need to understand/investigate what is the proper microenvironment for resident stem cells preservation and differentiation. First, in order to investigate the stimuli that drive stem cells into myogenesis, one should identify the interstitial cells that might provide the proper environment for the maintenance of a potential interstitial stem cell population and for their directed differentiation during muscle regeneration.

Many physical and soluble signals are needed in order to maintain a balance between proliferation and differentiation in order to restore normal tissue architecture.

Such signals derive either from muscle cells after injury, or from inflammatory or stromal cells, at their turn influenced by injury.

### 5.1. Inflammatory Cells and Cytokines (Paracrine Function)

The inflammatory response following acute injury undergoes a series of carefully regulated steps to efficiently recover tissue homeostasis.

During inflammatory stage different classes of leukocytes will be sequentially recruited by the sarcoplasmic proteins released by myofibre necrosis and they will constantly play a role throughout the entire process. Neutrophils are the first to come, followed by monocytes that will become macrophages as soon as they reach the muscle interstitium.

Recent studies demonstrated that inflammation promotes injury, but equally the inflammatory response is critical to skeletal muscle regeneration. However, the underlying molecular mechanisms still remain largely elusive.

Infiltrated leukocytes and macrophages, besides removing of necrotic debris, release a large array of growth factors and cytokines involved in attracting myogenic stem cells and promoting angiogenesis.

#### 5.1.1. Neutrophils

The infiltrating neutrophils contribute to sarcolemma lysis, and this process seems to be mediated by myeloperoxidase (MPO). Muscle injury typically induces a local increase in MPO activity that reflects neutrophil activation, extravasation, and cytotoxicity. The number of neutrophils increases two hours after the acute injury, but they become undetectable 3 days later. Their involvement in striated muscle regeneration or remodelling relies on the oxidative or proteolytic modification of damaged tissue, to allow phagocytosis of debris [97]. Recent studies showed that muscle regeneration is slower after toxic injury preceded by intraperitoneal injections of antisera to neutrophils and monocytes in order to deplete the phagocytes [128].

#### 5.1.2. Macrophages

Macrophages represent the most important cell population throughout the first days after injury, being responsible not only for the removal of cellular debris and apoptotic cells but also for the release of specific cytokines.

Recent data estimated the evolution of the numbers of macrophages after acute injury in wild-type mice skeletal muscles. Apparently they peaked after 3 days after injury, slowly decreased up to 7 days, and returned to baseline after 2 weeks following injury [34].

During the first 24 hours, the injured muscle will recruit CX3CR1lo/Ly-6C+ blood monocytes [129]. Within the muscle they release proinflammatory cytokines (TNF*α*, INF*γ*, and IL1*β*) that amplify tissue damage [101], by a NO-mediated mechanism generated by inducible nitric oxide synthase (iNOS) [130]. These cells were named CD68+ inflammatory (*M1*) cells and they are activated by INF*γ*. In the next days, as the phagocytosis ends, they will suffer a phenotypic and functional switch by the intervention of a set of cytokines IL-4, IL-10, and IL-13, to the activated (*M2*) cells, with increased expression of the mannose receptor CD206. Such cells were found near the regenerating muscle fibres. There are three subclasses of M2 cells, based on functional and molecular features: M2a macrophages, CD68−/CD163−/CD206+ induced by IL-4 and IL-13, functioning in advanced stages of healing and tissue repair; M2b macrophages that release anti-inflammatory cytokines like IL10; and M2c macrophages, CD68−/CD163+/CD206+, induced by IL-10. They can inactivate the M1 phenotype by IL-4 and IL-10 production to reduce muscle damage and promote myogenic differentiation, myofibre growth, and membrane repair [101].

According to recent findings, macrophages play a central role in controlling skeletal muscle regeneration by supporting muscle healing through remodelling the extracellular matrix and angiogenesis [131]. Both human and animal studies suggest that such cells have also a direct influence on other immune cells and progenitor/stem cells proliferation and migration and delay in differentiation of satellite cells by secretion of various cytokines and growth factors [49].

Recently,* IL-10* was proposed as a key player in survival and differentiation of transplanted mesoangioblasts, both* in vitro* and* in vivo*. IL-10 is actively produced by M2 macrophages and they represent the major source of IL-10 as long as their depletion restricts the expression of this cytokine [116].

Many studies indicate that* TNF*α*,* another proinflammatory cytokine, not only has a role in activating leukocytes and adhesion molecules on endothelial cells and controlling the synthesis of other cytokines and receptors [67], but affects muscle repair as well [132]. TNF*α* level rises in skeletal muscle with a peak at 24 hours after crush injury especially in activated leukocytes and macrophages [67]. However, TNF*α* levels remain raised for 2 weeks after acute injury, even though inflammatory phase declines, and this rise is paralleled by an increased expression of type I TNF*α* receptor in injured muscle fibres [133].

It has been equally demonstrated that TNF*α* activates satellite cells to enter the cell cycle and accelerates G_1_-to-S phase transition [104]. Studies on TNF-*α* null mutants showed lower level expression of proliferation and early differentiation transcription factors (MyoD and MEF-2) than wild-type after acute injury [101].

On the other hand, TNF*α* also provides inhibitory effects during transition from early differentiation to terminal differentiation by expression of muscle-specific genes and myotube formation. Such influences are carried on through NF-*κ*B activation that will furthermore stimulate the production of other inflammatory cytokines such as* CCL2* and* IL6* [134]. However, by activation of p38MAPK, an alternate signalling pathway, TNF*α* together with* IL1*, synthetized by invading macrophages as well, can promote muscle differentiation [133, 135].

More recent studies demonstrated that high mobility group box 1* (HMGB1)* and* TNF*α** secreted by local M1 cells and of* MMP-9* by M2 cells are responsible for recruiting myogenic stem cells, including vessel-associated stem cells (mesoangioblasts), which have been shown to preferentially home to injured skeletal muscle [49].

Even though so many studies proved multiple effects of TNF*α*, no defects in muscle repair or regeneration were reported in TNF*α* null mutant mice after muscle crush injury. One possible explanation relies on a compensatory mechanism based on cytokine system redundancy [67].

Another proinflammatory cytokine with similar effects on muscle regeneration is* IL-6*. Synthesis of IL-6 is stimulated in macrophages by the NF-*κ*B mechanism which also mediates the positive effects on myoblasts proliferation and transition to early differentiation and the inhibitory effects on terminal differentiation and cell fusion [101].

*In vitro* studies suggested the existence of a crosstalk between p38 MAPK and NF-*κ*B signalling pathways with activation of p38 preceding that of NF-*κ*B during myoblasts differentiation and the induction of IL-6 as an effector of the myogenic mechanism [136].

*IFN-*γ* expression* was not only found to correlate with the accumulation of macrophages after acute muscle injuries, but it was also expressed in T-cells, natural killer cells, and myoblasts. Functional* in vivo* studies with blocking antibodies or INF-*γ* null mice showed impaired muscle regeneration due to restricted cell proliferation and accelerated fibrosis. Those effects can be explained not only by impaired macrophage activation but also by direct influence on myogenic cells.* In vitro* testing showed that, by blocking INF-*γ* receptor on C2C12 muscle cell line, their proliferation and fusion are greatly reduced [137].

Macrophages also produce* TGF*β**. TGF*β* levels increase rapidly upon acute injuries in parallel with macrophage infiltration. It functions as a potent inhibitor of myogenic differentiation and promoter of fibrosis [88].

Functional studies on regenerating muscle based on neutralization of TGF-*β*1* in vivo* have been shown to lead to a reduction of the diameter of regenerating myofibres [129].

*CC chemokines* like MCP-1 (CCL2), MIP-1**α** (CCL3), and MIP-1**β**(CCL4) are greatly upregulated following experimental muscle injury as they were shown to induce myoblasts proliferation.

*MCP-1* a member of the CC chemokine family and the receptor CC chemokine receptor 2 (*CCR2*) are expressed primarily not only on macrophages [71] but also on fibroblasts, smooth muscle cells, or endothelial cells, even though some studies suggest conflicting results [34, 69].

Recovery of muscle structure after acute injury is significantly impaired in mice lacking its primary receptor, CCR2 [71], primarily by limited recruitment of monocytes/macrophages to damaged muscles [34].

MCP-1 is a chemokine known to be majorly involved in macrophage recruitment and activation. The absence of MCP-1 in MCP-1−/− mice was followed by impaired macrophage recruitment, muscle regeneration, and adipocyte accumulation but not as much as in CCR2−/− mice [71].

Recent data showed that MCP-1 also promotes angiogenesis [138]. However, MCP-1−/− mice presented a capillary density comparable to wild-type, suggesting that fewer macrophages are involved in angiogenesis than in muscle regeneration [71]. MCP-1 expression was increased in ischemic muscle after femoral artery excision, where angiogenesis and muscle regeneration occurred, but not in the nonischemic muscles, where collateral arteries formed by arteriogenesis [139].

Previous studies performed* in vitro* suggested that the angiogenic effect is either based on TGF*β* by recruiting vascular smooth muscle cells and mesenchymal cells toward endothelial cells or by upregulating hypoxia-*inducible factor 1α* and subsequent VEGF-A production [140]. In wild-type mice tissue VEGF level decreased after cardiotoxin-induced injury followed by necrosis and inflammation and it was restored within 7 days but it remained significantly reduced in mice lacking the CCR2 until day 21 [68].

Even if macrophages produce* VEGF*, they are not the major source during skeletal muscle regeneration in acute injuries as long as VEGF level did not correlate with the 3rd day peak in macrophage infiltration. Maximum capillary density was obtained only when VEGF level was restored to baseline and then decreased to control when fibres muscle reached normal cross-sectional area. Such results demonstrate that restoration of tissue VEGF levels is a CCR2-dependent process during skeletal muscle regeneration.

CCR2 is expressed only by proinflammatory M1 macrophages that are initially recruited to injured tissues while anti-inflammatory M2 macrophages do not express CCR2 and seem to be important in angiogenesis [71].

Recently, it has been demonstrated that intramuscular F4/80 macrophage in injured muscle is the major cellular source of* IGF-1* [34]. Many studies have proven increased IGF-1 mRNA levels during muscle regeneration.* In vitro* studies also showed that IGF-1 stimulates myoblast proliferation and differentiation.

Studies performed on CCR2−/− mice with impaired recruitment of circulating monocytes proved that the expression of IGF-1 was significantly higher in wild-type mice correlated with macrophage infiltration—upregulation at day 1 that peaks at day 3 and gradually falls toward day 21. When compared with intraperitoneal macrophages during acute peritonitis, the level of IGF-I produced by intramuscular macrophages was considerably higher.

It was previously demonstrated that local expression of IGF-1 speeded up the regeneration of skeletal muscle after injury by activating satellite cells, increasing the recruitment of other stem cells, and improving the survival of motor neurons. By modulating the inflammatory response and limiting fibrosis, IGF-1 maintains the balance between inflammation and connective tissue remodelling [141].

Macrophages also express urokinase-type plasminogen activator* (uPA)*. Studies on uPA null mice have shown impaired macrophage accumulation and muscle regeneration. Novak et al. [142] demonstrated that transgenic mice expressing only macrophage-derived uPA obtained by cross-breeding mice overexpressing macrophage-derived uPA and uPA null showed normal levels of macrophage accumulation, angiogenesis, and tissue repair after acute injuries. One of the suggested mechanisms includes the proteolytic activation of hepatocyte growth factor that promotes myoblast proliferation [143].

Besides the paracrine function defined by secretion of this large array of soluble factors, macrophages have been shown to establish direct cell-cell contacts with myogenic cells* in vitro* [92]. Such studies demonstrated that macrophages can rescue myogenic cells from apoptosis. Normally, adult muscle fibres are resistant to proapoptotic signals, but such signals can function in myogenic cells during muscle regeneration in order to control the number and the quality of the newly formed myofibres. This study evaluated the expression and function of four prosurvival cell-cell adhesion molecular systems on macrophages and myogenic cells* in vitro* and* in vivo*—VCAM-1-VLA-4, ICAM-1-LFA-1, PECAM-1-PECAM-1, and CX3CL1-CX3CR1 to prove that macrophages rescue differentiated myotubes from apoptosis probably in order to shelter them until they anchor themselves in the growing extracellular matrix.

#### 5.1.3. Mast Cells

There are also reports on* mast cells* potential role in normal skeletal muscle regeneration [95]. In the normal tissue, the number of mast cells is very low. One study used a mild injury model such as saline injection to prove the recruitment of mast cells from the circulation in the injected muscle, 8 hours after the injection.

Mast cells release not only histamine and proteases such as* chymase* and* tryptase* but also proinflammatory cytokines such as* TNF*α*, IL-1, and IL-6*, which rise early after acute damage.

It appears that mast cells are involved in activation and proliferation of endothelial cells and smooth muscle cells and by tryptase release they can also activate fibroblasts through the cleavage of protease-activated receptor-2 (PAR-2). Recent studies demonstrated the activation and expression of this receptor in rat skeletal muscle that promoted myoblast proliferation* in vitro* [93].* In vivo*, mast cells can indirectly influence myoblast proliferation by modulating macrophage recruitment [94].

### 5.2. Stromal Cells

Stromal cells represent the most flexible player in the myogenic cell behaviour modulation due to their distribution and high mobility in the interstitial tissue. Based on recent evidences, some distinct populations have been described and they seem to be involved in adjusting stem cell quiescence, self-renewal, differentiation, and apoptosis. To what extent those populations overlap or if they represent distinct cell populations was only marginally approached. Some of such studies, as in the case of stem cell populations, have been performed* in vitro*, after FACS isolation, so their distribution is not clear yet.

#### 5.2.1. Telocytes

Recently, we demonstrated the presence of a new type of interstitial cell in adult skeletal muscle tissue, the telocyte* (TC)* [90]. At present, the exact role of TCs is still under investigation, but based on morphologic assessment assumptions have been formulated. TCs seem to connect cells of a variety of types present in the muscular tissue by their long, thin cell projections, named telopodes. Due to their long-distance connections, TCs might play an essential part in integrating signals for skeletal muscle regulation, remodelling, and regeneration. TCs might provide not only paracrine signalling by releasing growth factors but also a framework for myogenic progenitor cells guidance during migration and differentiation after activation. Electron microscopy on normal skeletal muscle samples showed that TCs are often located close to blood vessels, nerve endings, and satellite cells or even putative progenitor cells and therefore they might offer guidance and paracrine support within the stem cell niche.

#### 5.2.2. Tcf4 Positive Fibroblasts

Recently a new regulatory mechanism of myogenesis was described in mouse tissue. This mechanism involves* Tcf4 positive fibroblasts* that reside in the muscle connective tissue sheaths [86]. Using Tcf4-GFP-Cre mice, the study demonstrated that muscle fibroblasts regulate muscle fibre type development and maturation.

Subsequent studies showed that the reciprocal interaction of such cells with the main stem cells in adult muscle—the satellite cells—contributes to efficient muscle regeneration. Experimental ablation of satellite cells not only impairs muscle regeneration but also interferes with fibroblasts function leading to an increase in connective tissue. By ablating Tcf4+ fibroblasts, satellite cells differentiate prematurely and the regenerated myofibres are smaller [87].

#### 5.2.3. Mesenchymal Progenitors (MPs)

Other quiescent cell populations residing in muscle interstitium that are activated after tissue damage were called fibro/adipogenic progenitors (*FAPs*) [83]. FAPs were recently identified as mesenchymal progenitors expressing PDGFR*α*, Sca-1, and CD34 distinct from canonical satellite cells that, upon activation, do not generate myofibres. Similar studies showed that PDGFR*α*+ mesenchymal progenitors can also differentiate toward osteoblastic and smooth muscle-like cells [10]. Apparently, their number increases in regenerating muscles without differentiation [83, 144]; their differentiation appears to be inhibited by direct contact with regenerating myofibres that most likely provide a local microenvironment that maintains their undifferentiated status [10, 145]. In cocultivation experiments, they were shown to stimulate the differentiation of primary myogenic progenitors, so they have been assumed to represent a transient source of prodifferentiation signals for proliferating myogenic progenitors during muscle regeneration. In degenerating skeletal muscles, FAPs are the major contributors to ectopic fat cell formation. It was not yet investigated if those adipocytes inhibit myogenesis, as has been suggested to happen in other tissues. It is also not clear yet if FAPs represent a completely distinct population or they are only a phonotypical variant of the adipocyte progenitors described in the white adipose tissue [144]. Based on shared PDGFR*α* and Sca-1 expression and the adipogenic potential, the same question could be raised regarding the recently described* Tie2+ skeletogenic progenitors* [89]. Such cells were identified in the skeletal muscle interstitium and distributed around blood vessels, and they represent the major source for heterotopic ossification due to their high osteogenic potential.

So far, such data could only imply the presence of a rather heterogeneous mesenchymal progenitor cell population with different subtypes selected by the microenvironment provided by specific conditions of the muscle tissue.

#### 5.2.4. Skeletal Muscle-Derived Fibroblast

Another study reported the selection in culture of a* skeletal muscle-derived fibroblast* population that expressed SDF-1. The conditioned media collected from such cultures chemoattracted CXCR4 positive murine satellite cells. Neutralization of SDF-1 decreased this effect; therefore the authors concluded that the process involved SDF-1/CXCR4+ signalling pathway [24]. Either those muscle-derived fibroblasts represent a distinct interstitial cell population or they overlap with the ones previously presented was not investigated up to now.

There is a clear tendency to include all connective tissue cells into the ambiguous family of fibroblasts, but based on the results presented so far a general reassessment of interstitial cells, from morphological, phenotypical, and differentiation points of view, would be required, probably not only for the skeletal muscle connective tissue.

### 5.3. Vascular Cells

During regeneration, angiogenesis and myogenesis are coordinated processes. Upon injury, destruction of vascular network allows a direct crosstalk between exposed vascular cells and potential myogenic progenitors: angiogenesis is an absolute requirement for tissue repair and, on the other hand, vascular cells provide an extensive array of potent soluble factors.

*Endothelial Cells*.  Endothelial cells stimulate growth of satellite cells through the secretion of a variety of* growth factors*, including IGF-1, VEGF, FGF-2, PDGF-BB, and HGF [33]. They also secrete* cytokines* like IL-6, IL-8, and TNF*α* [29].

Acute injuries with necrosis/inflammation will also lead to endothelial cell destruction and subsequent decrease in soluble factors synthetized by this cell population, such as VEGF [68]. VEGF levels will be restored in conjunction with muscle regeneration and capillary network reconstruction in order to support myoblast growth and survival.

Endothelial cells also produce MCP-1 which promotes recruitment and activation of macrophages [69] (see above).

*Periendothelial cells* are represented by* smooth muscle cells* or*pericytes* and* endomysial interstitial cells* that stabilize the blood vessel. It was demonstrated, both* in vitro* and* in vivo*, that such cells promote the return to quiescence of muscle progenitors by* angiopoietin-1* release, through Angiopoietin-1/Tie-2 signalling system. Angiopietin-1 inhibits growth, proliferation, and differentiation of myogenic cells. Tie-2 expression increases as myogenic cells return to quiescence.* In vivo* functional studies with blocking antibodies against Ang-1/Tie-2 drove satellite cells back into the cell cycle. Thus, the recovery of vascular integrity very probably becomes a signal for ending myogenesis [29].

### 5.4. Peripheral Nerve Cells

#### 5.4.1. Motor Neurons

Studies comparing muscle regeneration in different mouse injury models showed that myoblast differentiation and subsequent fusion occur more rapidly in myotoxic injuries than after crushing. The major difference between the two models is that not only blood vessels but also peripheral nerves are affected during skeletal muscle traumatic injuries. In order to obtain full regeneration, reinnervation is mandatory; otherwise it can lead to progressive muscle atrophy [146].

Reinnervation takes place starting from the surrounding, not damaged tissue, and accounts directly for the molecular profile of the skeletal muscle fibre generating either slow-twitch/oxidative or fast-twitch/glycolytic myofibres. Even though myofibre recovery from atrophy after innervation reestablishment was thought to rely on satellite cell incorporation, recent studies on mice proved that this connection does not directly stimulate satellite cells activation, as long as there was no significant difference between the reinnervated and contralateral muscle [147]. Moreover, this experimental approach proved that even though after reinnervation satellite cells are activated, expressing MyoD, they do not upregulate myogenin, as a differentiation marker.

Such results clearly suggest that the crosstalk between nerves and muscle fibres during muscle regeneration relies on other factors that accompany muscle and nerve injuries and can spread in other distant locations, either as soluble factors secreted by nonneuronal cells of the peripheral nerve, or even the cells themselves. This subject was scarcely approached up to now.

#### 5.4.2. Nonneuronal Cells

One nonneuronal source of soluble factors is represented by* Schwann cells* of adult peripheral nerves and such an example is ciliary neurotrophic factor* (CNTF)*. CNTF is abundantly released during muscle damaged accompanied by nerve injury. Previous studies suggested that CNTF administration during muscle regeneration favours myotube differentiation [78].

Recently it was proven that CNTF supports myogenic progenitor cell viability* in vitro* by the PI3-Akt signalling pathway [148].

As expected, also the CTNF receptor alpha (CTNFR*α*) expression increases after crushing injuries involving both muscle and nerve. However, according to a recent study CTNFR*α* is needed primarily for neuronal regeneration rather than regeneration of the muscle itself [149].

Such results clearly support the concept of nerve-muscle crosstalk following traumatic injuries.

Another neurotrophic factor that also promotes myoblasts viability is insulin-like growth factor-1 (IGF-1). One important source could be represented by Schwann cells as stated by a study on extraocular muscle force regulation. Apparently muscle cells preferentially extract IGF from the nerve than from the systemic source [150].

Upon transection, the expression levels of another neurotrophic factor, nerve growth factor (NGF), significantly increase in the peripheral nerve in all nonneuronal cells [151]. Nerve growth factor has been shown to stimulate myoblast proliferation and fusion* in vitro,* and also* in vivo*, but to a lesser extent [150].

NGF has been tested in other studies for modulating muscle-derived stem cell behaviour prior to transplantation. Apparently,* in vitro* direct stimulation reduces differentiation potential but increases the engraftment of MDSC upon transplantation into dystrophic mdx mice [152].

Even without nerve injury, there is a constant remodelling of the nerve terminal during degeneration and regeneration of the segment of the postsynaptic muscle fibre, as this connection is mandatory for functional recovery of the muscle tissue [153]. Studies on muscle fibre laser ablation and vital imaging proved that nerve terminal and Schwann cells remain in contact with the synaptic basal lamina. As muscle fibres regenerate, AchRs accumulate in the membrane to the synaptic sites. New junctions will be established by new extensions of the nerve terminal, guided by processes of terminal Schwann cells outside the area of the original contact.

## 6. Concluding Remarks

The reorganization of muscle extracellular matrix after injury is mandatory for providing the appropriate scaffold for the regenerated myofibres and their precise spatial organization.

The fine line between efficient tissue regeneration and scar formation relies on mechanisms that are still largely unknown. In the last few years, multiple resident cell populations that support muscle progenitor cell activation were taken into account when regeneration-therapy hypotheses were imagined. However, keeping these cell types balance and control is clearly the key for tissue homeostasis and efficient regeneration.

Connective tissue is one of the most abundant but also the most unpredictable type of tissues in terms of cellular and extracellular molecular composition that varies greatly according to mechanical and soluble stimuli. This tissue type not only has no problem in regenerating itself but also could help or completely impair, by scar formation, the function of neighbouring tissues after various organ injuries.

Development of new therapeutic targets requires broadening of the horizon of cellular therapy approaches beyond stem/progenitor cells toward a thorough investigation of the environmental changes and signals provided by other cell types in or surrounding the niche, taking into account the trivial interstitial cells.

## Figures and Tables

**Table 1:** Phenotypic synopsis of the cells found in skeletal muscle interstitium.

Cell types				Markers	Negative markers	Reference	Secreted molecules	Reference
Stem cells	Resident	Satellite cells^∗#^		Pax7, Pax3, Myf5, Barx2, M-cadherin, CD206, CCL2/CCR2, CTRs, CXCR4, c-Met, CD34, p75NTR, Tie-2, *α*7-intergin, *β*1-integrin, VECAM-1, syndecan-3 and syndecan-4, NCAM/CD56, caveolin-1, Nestin, SM/C-2.6, PW1, HMGB1	CD45, Sca-1, desmin, MyHC	[15, 20–41]		
		Nonspecified location	Muscle-derived side population cells (MSP cells)*	Abcg2, Sca-1, CD11, Gr-1, CD5	c-kit, CD43, CD45, CD34.	[8, 42, 43]		
			PICs^#^	PW1, Sca-1, CD34, vimentin	PDGFR*α*	[43, 44]		
			Slow-adhering stem cells (SASCs)*	Notch1, STAT3, Msx1, Pax3, MMP2		[45]		
			Sk-34 myoendothelial progenitors^#^	CD34, Sca-1, c-Met	CD45, CD14, CD31, CD49, CD144 Flk1, MyoD, myf-5, myf-6, myogenin, M-cadherin, Pax-3, Pax-7.	[46]		
		Vessel-associated	Mesoangioblasts (vessel-associated stem cells)*	E-selectin, *β*7, integrin, AlCAM, CD44, cav1, FGFR1, CXCR-4, TNF-R, TNFR 1a, IL-10R, IL-4R, RAGE, TLR4 (HMGB1 rec), CD13	CD34, CD45, CD117 CD31	[47–49]	HMGB1, VEGF, CXCL12/SDF1, CCL2, VEGF B, bFGF, FGF7, PDGF AA, HDGF	[49, 50]
			Myogenic endothelial cells (MECs)^#^	CD56, CD34, Pax7, von Willebrand factor, VE-cadherin/CD144, UEA-1 receptor	CD45	[51, 52]		
			Pericytes^∗#^	CCR2, Abcg2, Alkaline phosphatase, *α*SMA, desmin, NG2, aminopeptidase A/B, RGS5, CD146, PDGFR*α* and *β*, NG2	vWF, CD31, CD34, CD144, CD45, CD56, Pax7	[52–60]	Ang 1	[29]
			Adventitial cells (ACs)^#^	CD34, Sca-1, Flk1, CD140b	c-kit, CD31, CD146, CD45	[61, 62]		
	Blood-derived		AC133+ cells	CD133, CD34, CD90, CD45		[63]		
			Bone marrow-derived SP cells	c-kit, CD43, CD45, Sca-1	B220, Mac-1, Gr-1, CD4, CD5, CD8, CD34	[42]		

Interstitial cells	Vascular cells		Endothelial cells	von Willebrand factor, CD146, CD31, CD34, CD144, CD105, Abcg2, Tie-2	CCR2	[52, 54, 56, 64–66]	MCP-1, TNF*α*, VEGF, IGF-1, FGF-2, PDGF-BB, HGF, IL-6, IL-8, TNF*α*	[29, 33, 67–69]
			Smooth muscle cells	SMA, CCR2, *α*-actin, caldesmon, calponin		[59, 70, 71]	Ang 1	[29]
	Nerve cells		Schwann cells	NCAM/CD56, nestin S-100, GFAP, Krox24	cytokeratin	[72–77]	CTNF, IGF-1, NGF	[78]
					
			Perineurial cells	Epithelial membrane antigen (EMA) GLUT-1, claudin-1, ZO-1, connexins, occludin, vinculin, talin, desmin, titin, spectrin, CD34, VE-cadherin, AQP-1, tenascin-C	cytokeratin S-100, CD57, neurofilaments	[74, 79, 80]		
			Neurons (axon)	Nf200, *β*III tubulin		[76]	Neuregulin, calcitonin gene related peptide (CGRP)	[76]
	Resident cells in muscle connective tissue sheaths		Fibroblasts	CCR2, vimentin, procollagen, ET-TR, FSP-1, SMA		[60, 81–85]	PDGF AA, MCP-1, TNF*α*, Ang-1	[29, 50, 67, 69]
			Tcf4+ fibroblasts^#^	Tcf4, PDGFR*α*	Pax7, MyoD, F4/80	[86, 87]		
			SDF-1+ muscle-derived fibroblasts*				SDF-1	[24]
			MPs					
			FAPs^∗#^	Sca-1, PDGFR*α*, CD34	CD45, CD31, *α*7 integrin	[10, 56, 83, 88]	IL6	[83]
			PDGFR*α*+^∗#^	PDGFR*α*, vimentin	NG2, SMA, Pax7, M-cadherin	[10]		
			Tie2+ skeletogenic progenitors^∗#^	Tie2, Sca-1, PDFGR*α*, CD29, CD34	CD31, CD45, CD11b, CXCR4, c-kit, NG2	[89]		
			Telocytes (TCs)^∗#^	c-kit, CD34, vimentin, PDGFR*α*/*β*		[90, 91]	VEGF	[91]
	Blood-derived connective tissue cells		Macrophages M1	CD68, CD86, CD40, CD14, MHC I/II, CCR2		[49, 71]	TNF*α*, IL1*β*, TNF*α*, IL-6, TNF-*α*, IL- 1*β*, IL-12, IFN-*γ*, NO, VEGF, HMGB1, CCL2, MCP-1	[49, 67]
			Macrophages M2	CD163, CD36, CD163, CD206, MHC I/II, CD14, *α*V-integrin, *α*V*β*3I, VCAM-1, membrane-bound CX3CL1, ICAM-1 PECAM-1		[49, 92]	IGF1, IL10, MMP-9, IL10 and Low: IL-6, TNF-*α*, IL- 1*β*, IFN-*γ*	[49]
			Mast cells	c-kit, mast cell triptase		[93, 94]	TNF*α*, histamine, chymase, tryptase, IL-1, IL-6	[67, 93, 95]
			Polimorphonuclear	CXCR2, CD11b, CD45		[96]	MPO	[97]

	Myoblasts			Myogenin, Pax3 (cycling myoblasts), *α*-actin, myosin heavy chain, CCR1 and CCR5, CCR2. gp130 and LIFR, NCAM/CD56, VLA-4, CX3CR1, LFA-1 and PECAM-1, Sca-1, Tie-2, p75NTR, desmin, Myf5, HMGB1, TLR4, nestin	Pax7, CCR2	[28, 29, 34, 39, 73, 78, 88, 92, 98–103]	TNF*α*, INF*γ*, VEGF, Ang-1 G-CSF/G-CSFR	[104, 105]

	Myocytes/Myotubes			Myogenin, MRF4, CNTFR, NCAM/CD56, nestin, RAGE		[34, 73, 78, 102, 103]	TNF*α*	[104]

*In vitro, ^#^ *in situ*.

**Table 2:** Satellite cell markers.

Early myogenic markers	Pax7	Paired domain transcription factors	[15]
	Pax3	Paired domain transcription factors	[20]
	Myf5	Myogenic regulatory factor	[21]
	M-cadherin	Cell adhesion protein	[8, 23]

Transcription factors	Msx1	Homeobox transcription factor	[45, 99]
	Barx2	Homeobox transcription factor	[22]

Receptors	CD206	Mannose receptor	[101]
	CCL2/CCR2	CC chemokine MCP-1	[101]
	CTRs	Calcitonin receptors	[106]
	CXCR4	Chemokine receptor	[24]
	c-Met	Tyrosine kinase receptor for HGF	[25]
	p75NTR	Neurotrophin receptor	[28]
	Tie-2	Tyrosine kinase receptor for angiopoietin 1	[29]

Adhesion molecules	CD34	Single-pass transmembrane sialomucin	[8, 26, 27]
	*α*7-intergin	Cell surface attachment receptor	[107]
	*β*1- integrin	Cell surface attachment receptor	[40]
	VECAM-1	Adhesion molecule	[41]
	Syndecan-3/4	Transmembrane heparan sulfate proteoglycans	[32, 108]
	NCAM/CD56	Neural cell adhesion molecule	[33, 34]

Other	caveolin-1	Membrane protein	[31, 35]
	lamin A/C and emerin	Nuclear envelope proteins	[31]
	Nestin	Intermediate filament protein	[109]
	SM/C-2.6		[36]
	PW1	Cell stress mediator	[37]
	HMGB1	High mobility group box 1 protein	[103]

## References

[1] Huard J, Li Y, Fu FH (2002). Muscle injuries and repair: current trends in research. *Journal of Bone and Joint Surgery A*.

[2] Stratos I, Rotter R, Eipel C, Mittlmeier T, Vollmar B (2007). Granulocyte-colony stimulating factor enhances muscle proliferation and strength following skeletal muscle injury in rats. *Journal of Applied Physiology*.

[3] Mauro A (1961). Satellite cell of skeletal muscle fibers. *The Journal of Biophysical and Biochemical Cytology*.

[4] Schultz E, Gibson MC, Champion T (1978). Satellite cells are mitotically quiescent in mature mouse muscle: an EM and radioautographic study. *Journal of Experimental Zoology*.

[5] Ontell M, Feng KC, Klueber K, Dunn RF, Taylor F (1984). Myosatellite cells, growth, and regeneration in murine dystrophic muscle: a quantitative study. *Anatomical Record*.

[6] Zammit PS (2008). All muscle satellite cells are equal, but are some more equal than others?. *Journal of Cell Science*.

[7] LaBarge MA, Blau HM (2002). Biological progression from adult bone marrow to mononucleate muscle stem cell to multinucleate muscle fiber in response to injury. *Cell*.

[8] Asakura A, Seale P, Girgis-Gabardo A, Rudnicki MA (2002). Myogenic specification of side population cells in skeletal muscle. *Journal of Cell Biology*.

[9] Asakura A, Komaki M, Rudnicki MA (2001). Muscle satellite cells are multipotential stem cells that exhibit myogenic, osteogenic, and adipogenic differentiation. *Differentiation*.

[10] Uezumi A, Fukada S, Yamamoto N, Takeda S, Tsuchida K (2010). Mesenchymal progenitors distinct from satellite cells contribute to ectopic fat cell formation in skeletal muscle. *Nature Cell Biology*.

[11] Oishi T, Uezumi A, Kanaji A (2013). Osteogenic differentiation capacity of human skeletal muscle-derived progenitor cells. *PLoS ONE*.

[12] Relaix F, Zammit PS (2012). Satellite cells are essential for skeletal muscle regeneration: the cell on the edge returns centre stage. *Development*.

[13] Collins CA, Olsen I, Zammit PS (2005). Stem cell function, self-renewal, and behavioral heterogeneity of cells from the adult muscle satellite cell niche. *Cell*.

[14] Yin H, Price F, Rudnicki MA (2013). Satellite cells and the muscle stem cell niche. *Physiological Reviews*.

[15] Seale P, Sabourin LA, Girgis-Gabardo A, Mansouri A, Gruss P, Rudnicki MA (2000). Pax7 is required for the specification of myogenic satellite cells. *Cell*.

[16] Lepper C, Conway SJ, Fan C (2009). Adult satellite cells and embryonic muscle progenitors have distinct genetic requirements. *Nature*.

[17] Lepper C, Partridge TA, Fan C (2011). An absolute requirement for pax7-positive satellite cells in acute injury-induced skeletal muscle regeneration. *Development*.

[18] Gunther S, Kim J, Kostin S (2013). Myf5-positive satellite cells contribute to Pax7-dependent long-term maintenance of adult muscle stem cells. *Cell Stem Cell*.

[19] von Maltzahn J, Jones AE, Parks RJ, Rudnicki MA (2013). Pax7 is critical for the normal function of satellite cells in adult skeletal muscle. *Proceedings of the National Academy of Sciences of the United States of America*.

[20] Buckingham M, Bajard L, Chang T (2003). The formation of skeletal muscle: from somite to limb. *Journal of Anatomy*.

[21] Cornelison DDW, Wold BJ (1997). Single-cell analysis of regulatory gene expression in quiescent and activated mouse skeletal muscle satellite cells. *Developmental Biology*.

[22] Meech R, Gonzalez KN, Barro M (2012). Barx2 is expressed in satellite cells and is required for normal muscle growth and regeneration. *Stem Cells*.

[23] Irintchev A, Zeschnigk M, Starzinski-Powitz A, Wernig A (1994). Expression pattern of M-cadherin in normal, denervated, and regenerating mouse muscles. *Developmental Dynamics*.

[24] Ratajczak MZ, Majka M, Kucia M (2003). Expression of functional CXCR4 by muscle satellite cells and secretion of SDF-1 by muscle-derived fibroblasts is associated with the presence of both muscle progenitors in bone marrow and hematopoietic stem/progenitor cells in muscles. *Stem Cells*.

[25] Allen RE, Sheehan SM, Taylor RG, Kendall TL, Rice GM (1995). Hepatocyte growth factor activates quiescent skeletal muscle satellite cells in vitro. *Journal of Cellular Physiology*.

[26] Beauchamp JR, Heslop L, Yu DSW (2000). Expression of CD34 and Myf5 defines the majority of quiescent adult skeletal muscle satellite cells. *Journal of Cell Biology*.

[27] Alfaro LAS, Dick SA, Siegel AL (2011). CD34 promotes satellite cell motility and entry into proliferation to facilitate efficient skeletal muscle regeneration. *Stem Cells*.

[28] Colombo E, Romaggi S, Medico E (2011). Human neurotrophin receptor p75NTR defines differentiation-oriented skeletal muscle precursor cells: implications for muscle regeneration. *Journal of Neuropathology and Experimental Neurology*.

[29] Abou-Khalil R, Mounier R, Chazaud B (2010). Regulation of myogenic stem cell behavior by vessel cells: the “ménage à trois” of satellite cells, periendothelial cells and endothelial cells. *Cell Cycle*.

[30] Burkin DJ, Kaufman SJ (1999). The *α*7*β*1 integrin in muscle development and disease. *Cell and Tissue Research*.

[31] Gnocchi VF, White RB, Ono Y, Ellis JA, Zammit PS (2009). Further characterisation of the molecular signature of quiescent and activated mouse muscle satellite cells. *PLoS ONE*.

[32] Cornelison DDW, Filla MS, Stanley HM, Rapraeger AC, Olwin BB (2001). Syndecan-3 and syndecan-4 specifically mark skeletal muscle satellite cells and are implicated in satellite cell maintenance and muscle regeneration. *Developmental Biology*.

[33] Chiristov C, Chrétien F, Abou-Khalil R (2007). Muscle satellite cells and endothelial cells: close neighbors and privileged partners. *Molecular Biology of the Cell*.

[34] Lu H, Huang D, Saederup N, Charo IF, Ransohoff RM, Zhou L (2011). Macrophages recruited via CCR2 produce insulin-like growth factor-1 to repair acute skeletal muscle injury. *The FASEB Journal*.

[35] ten Broek RW, Grefte S, von den Hoff JW (2010). Regulatory factors and cell populations involved in skeletal muscle regeneration. *Journal of Cellular Physiology*.

[36] Fukada S, Higuchi S, Segawa M (2004). Purification and cell-surface marker characterization of quiescent satellite cells from murine skeletal muscle by a novel monoclonal antibody. *Experimental Cell Research*.

[37] Mitchell KJ, Pannérec A, Cadot B (2010). Identification and characterization of a non-satellite cell muscle resident progenitor during postnatal development. *Nature Cell Biology*.

[38] Anderson JE (2006). The satellite cell as a companion in skeletal muscle plasticity: currency, conveyance, clue, connector and colander. *Journal of Experimental Biology*.

[39] Conboy IM, Rando TA (2002). The regulation of Notch signaling controls satellite cell activation and cell fate determination in postnatal myogenesis. *Developmental Cell*.

[40] Cerletti M, Jurga S, Witczak CA (2008). Highly efficient, functional engraftment of skeletal muscle stem cells in dystrophic muscles. *Cell*.

[41] Jesse TL, LaChance R, Iademarco MF, Dean DC (1998). Interferon regulatory factor-2 is a transcriptional activator in muscle where it regulates expression of vascular cell adhesion molecule-1. *Journal of Cell Biology*.

[42] Gussoni E, Soneoka Y, Strickland CD (1999). Dystrophin expression in the mdx mouse restored by stem cell transplantation. *Nature*.

[43] Moresi V, Pristerà A, Scicchitano BM (2008). Tumor necrosis factor-*α* inhibition of skeletal muscle regeneration is mediated by a caspase-dependent stem cell response. *Stem Cells*.

[44] Pannerec A, Formicola L, Besson V, Marazzi G, Sassoon DA (2013). Defining skeletal muscle resident progenitors and their cell fate potentials. *Development*.

[45] Mu X, Xiang G, Rathbone CR (2011). Slow-adhering stem cells derived from injured skeletal muscle have improved regenerative capacity. *The American Journal of Pathology*.

[46] Tamaki T, Akatsuka A, Ando K (2002). Identification of myogenic-endothelial progenitor cells in the interstitial spaces of skeletal muscle. *Journal of Cell Biology*.

[47] Galvez BG, Sampaolesi M, Brunelli S (2006). Complete repair of dystrophic skeletal muscle by mesoangioblasts with enhanced migration ability. *Journal of Cell Biology*.

[48] Sampaolesi M, Blot S, D’Antona G (2006). Mesoangioblast stem cells ameliorate muscle function in dystrophic dogs. *Nature*.

[49] Lolmede K, Campana L, Vezzoli M (2009). Inflammatory and alternatively activated human macrophages attract vessel-associated stem cells, relying on separate HMGB1- and MMP-9-dependent pathways. *Journal of Leukocyte Biology*.

[50] Galli D, Innocenzi A, Staszewsky L (2005). Mesoangioblasts, vessel-associated multipotent stem cells, repair the infarcted heart by multiple cellular mechanisms: a comparison with bone marrow progenitors, fibroblasts, and endothelial cells. *Arteriosclerosis, Thrombosis, and Vascular Biology*.

[51] Zheng B, Cao B, Crisan M (2007). Prospective identification of myogenic endothelial cells in human skeletal muscle. *Nature Biotechnology*.

[52] Chen C, Corselli M, Péault B, Huard J (2012). Human blood-vessel-derived stem cells for tissue repair and regeneration. *Journal of Biomedicine and Biotechnology*.

[53] Armulik A, Abramsson A, Betsholtz C (2005). Endothelial/pericyte interactions. *Circulation Research*.

[54] Bagley RG, Weber W, Rouleau C, Teicher BA (2005). Pericytes and endothelial precursor cells: cellular interactions and contributions to malignancy. *Cancer Research*.

[55] Dellavalle A, Sampaolesi M, Tonlorenzi R (2007). Pericytes of human skeletal muscle are myogenic precursors distinct from satellite cells. *Nature Cell Biology*.

[56] Doyle MJ, Zhou S, Tanaka KK (2011). Abcg2 labels multiple cell types in skeletal muscle and participates in muscle regeneration. *Journal of Cell Biology*.

[57] Crisan M, Yap S, Casteilla L (2008). A perivascular origin for mesenchymal stem cells in multiple human organs. *Cell Stem Cell*.

[58] Dellavalle A, Maroli G, Covarello D (2011). Pericytes resident in postnatal skeletal muscle differentiate into muscle fibres and generate satellite cells. *Nature Communications*.

[59] Traktuev DO, Merfeld-Clauss S, Li J (2008). A population of multipotent CD34-positive adipose stromal cells share pericyte and mesenchymal surface markers, reside in a periendothelial location, and stabilize endothelial networks. *Circulation Research*.

[60] Sun D, Martinez CO, Ochoa O (2009). Bone marrow-derived cell regulation of skeletal muscle regeneration. *The FASEB Journal*.

[61] Corselli M, Chen CW, Sun B (2012). The tunica adventitia of human arteries and veins as a source of mesenchymal stem cells. *Stem Cells and Development*.

[62] Majesky MW, Dong XR, Hoglund V, Daum G, Mahoney WM (2012). The adventitia: a progenitor cell niche for the vessel wall. *Cells Tissues Organs*.

[63] Torrente Y, Belicchi M, Sampaolesi M (2004). Human circulating AC133^+^ stem cells restore dystrophin expression and ameliorate function in dystrophic skeletal muscle. *The Journal of Clinical Investigation*.

[64] Zengin E, Chalajour F, Gehling UM (2006). Vascular wall resident progenitor cells: a source for postnatal vasculogenesis. *Development*.

[65] Duff SE, Li C, Garland JM, Kumar S (2003). CD105 is important for angiogenesis: evidence and potential applications. *The FASEB Journal*.

[66] Schrage A, Loddenkemper C, Erben U (2008). Murine CD146 is widely expressed on endothelial cells and is recognized by the monoclonal antibody ME-9F1. *Histochemistry and Cell Biology*.

[67] Collins RA, Grounds MD (2001). The role of tumor necrosis factor-alpha (TNF-*α*) in skeletal muscle regeneration: studies in TNF-*α*(-/-) and TNF-*α*(-/-)/LT-*α*(-/-) mice. *Journal of Histochemistry and Cytochemistry*.

[68] Ochoa O, Sun D, Reyes-Reyna SM (2007). Delayed angiogenesis and VEGF production in CCR2-/- mice during impaired skeletal muscle regeneration. *American Journal of Physiology—Regulatory Integrative and Comparative Physiology*.

[69] Deshmane SL, Kremlev S, Amini S, Sawaya BE (2009). Monocyte chemoattractant protein-1 (MCP-1): an overview. *Journal of Interferon and Cytokine Research*.

[70] Rensen SSM, Doevendans PAFM, van Eys GJJM (2007). Regulation and characteristics of vascular smooth muscle cell phenotypic diversity. *Netherlands Heart Journal*.

[71] Martinez CO, McHale MJ, Wells JT (2010). Regulation of skeletal muscle regeneration by CCR2-activating chemokines is directly related to macrophage recruitment. *American Journal of Physiology—Regulatory Integrative and Comparative Physiology*.

[72] Daniloff JK, Levi G, Grumet M (1986). Altered expression of neuronal cell adhesion molecules induced by nerve injury and repair. *Journal of Cell Biology*.

[73] Íková D, Soukup T, Mokrý J (2009). Nestin expression reflects formation, revascularization and reinnervation of new myofibers in regenerating rat hind limb skeletal muscles. *Cells Tissues Organs*.

[74] Perentes E, Nakagawa Y, Ross GW, Stanton C, Rubinstein LJ (1987). Expression of epithelial membrane antigen in perineurial cells and their derivatives. An immunohistochemical study with multiple markers. *Acta Neuropathologica*.

[75] Chen Y-G, Brushart TM (1998). The effect of denervated muscle and schwann cells on axon collateral sprouting. *Journal of Hand Surgery*.

[76] Webber C, Zochodne D (2010). The nerve regenerative microenvironment: early behavior and partnership of axons and Schwann cells. *Experimental Neurology*.

[77] Blake JA, Ziman MR (2013). The characterisation of Pax3 expressant cells in adult peripheral nerve. *PLoS ONE*.

[78] Kami K, Morikawa Y, Sekimoto M, Senba E (2000). Gene expression of receptors for IL-6, LIF, and CNTF in regenerating skeletal muscles. *Journal of Histochemistry and Cytochemistry*.

[79] Yamamoto M, Okui N, Tatebe M, Shinohara T, Hirata H (2011). Regeneration of the perineurium after microsurgical resection examined with immunolabeling for tenascin-C and alpha smooth muscle actin. *Journal of Anatomy*.

[80] Piña-Oviedo S, Ortiz-Hidalgo C (2008). The normal and neoplastic perineurium: a review. *Advances in Anatomic Pathology*.

[81] Goodpaster T, Legesse-Miller A, Hameed MR, Aisner SC, Randolph-Habecker J, Coller HA (2008). An immunohistochemical method for identifying fibroblasts in formalin-fixed, paraffin-embedded tissue. *Journal of Histochemistry and Cytochemistry*.

[82] Alt E, Yan Y, Gehmert S (2011). Fibroblasts share mesenchymal phenotypes with stem cells, but lack their differentiation and colony-forming potential. *Biology of the Cell*.

[83] Joe AWB, Yi L, Natarajan A (2010). Muscle injury activates resident fibro/adipogenic progenitors that facilitate myogenesis. *Nature Cell Biology*.

[84] Strutz F, Okada H, Lo CW (1995). Identification and characterization of a fibroblast marker: FSP1. *Journal of Cell Biology*.

[85] Brack AS, Conboy MJ, Roy S (2007). Increased Wnt signaling during aging alters muscle stem cell fate and increases fibrosis. *Science*.

[86] Mathew SJ, Hansen JM, Merrell AJ (2011). Connective tissue fibroblasts and Tcf4 regulate myogenesis. *Development*.

[87] Murphy MM, Lawson JA, Mathew SJ, Hutcheson DA, Kardon G (2011). Satellite cells, connective tissue fibroblasts and their interactions are crucial for muscle regeneration. *Development*.

[88] Long KK, Montano M, Pavlath GK (2011). Sca-1 is negatively regulated by TGF-*β*1 in myogenic cells. *The FASEB Journal*.

[89] Wosczyna MN, Biswas AA, Cogswell CA, Goldhamer DJ (2012). Multipotent progenitors resident in the skeletal muscle interstitium exhibit robust BMP-dependent osteogenic activity and mediate heterotopic ossification. *Journal of Bone and Mineral Research*.

[90] Popescu LM, Manole E, Şerboiu CS (2011). Identification of telocytes in skeletal muscle interstitium: implication for muscle regeneration. *Journal of Cellular and Molecular Medicine*.

[91] Suciu LC, Popescu BO, Kostin S, Popescu LM (2012). Platelet-derived growth factor receptor-beta-positive telocytes in skeletal muscle interstitium. *Journal of Cellular and Molecular Medicine*.

[92] Sonnet C, Lafuste P, Arnold L (2006). Human macrophages rescue myoblasts and myotubes from apoptosis through a set of adhesion molecular systems. *Journal of Cell Science*.

[93] Duchesne E, Tremblay M, Cote CH (2011). Mast cell tryptase stimulates myoblast proliferation, a mechanism relying on protease-activated receptor-2 and cyclooxygenase-2. *BMC Musculoskeletal Disorders*.

[94] Duchesne E, Bouchard P, Roussel MP, Cote CH (2013). Mast cells can regulate skeletal muscle cell proliferation by multiple mechanisms. *Muscle & Nerve*.

[95] Kharraz Y, Guerra J, Mann CJ, Serrano AL, Munoz-Canoves P (2013). Macrophage plasticity and the role of inflammation in skeletal muscle repair. *Mediators of Inflammation*.

[96] Warren GL, O’Farrell L, Summan M (2004). Role of CC chemokines in skeletal muscle functional restoration after injury. *American Journal of Physiology—Cell Physiology*.

[97] Nguyen HX, Lusis AJ, Tidball JG (2005). Null mutation of myeloperoxidase in mice prevents mechanical activation of neutrophil lysis of muscle cell membranes in vitro and in vivo. *Journal of Physiology*.

[98] Yahiaoui L, Gvozdic D, Danialou G, Mack M, Petrof BJ (2008). CC family chemokines directly regulate myoblast responses to skeletal muscle injury. *Journal of Physiology*.

[99] Chargé SBP, Rudnicki MA (2004). Cellular and molecular regulation of muscle regeneration. *Physiological Reviews*.

[100] Zammit PS, Golding JP, Nagata Y, Hudon V, Partridge TA, Beauchamp JR (2004). Muscle satellite cells adopt divergent fates: a mechanism for self-renewal?. *Journal of Cell Biology*.

[101] Tidball JG, Villalta SA (2010). Regulatory interactions between muscle and the immune system during muscle regeneration. *American Journal of Physiology—Regulatory Integrative and Comparative Physiology*.

[102] le Grand F, Rudnicki MA (2007). Skeletal muscle satellite cells and adult myogenesis. *Current Opinion in Cell Biology*.

[103] de Mori R, Straino S, di Carlo A (2007). Multiple effects of high mobility group box protein 1 in skeletal muscle regeneration. *Arteriosclerosis, Thrombosis, and Vascular Biology*.

[104] Li Y (2003). TNF-*α* is a mitogen in skeletal muscle. *American Journal of Physiology—Cell Physiology*.

[105] Hara M, Yuasa S, Shimoji K (2011). G-CSF influences mouse skeletal muscle development and regeneration by stimulating myoblast proliferation. *Journal of Experimental Medicine*.

[106] Fukada S, Uezumi A, Ikemoto M (2007). Molecular signature of quiescent satellite cells in adult skeletal muscle. *Stem Cells*.

[107] Sherwood RI, Christensen JL, Conboy IM (2004). Isolation of adult mouse myogenic progenitors: functional heterogeneity of cells within and engrafting skeletal muscle. *Cell*.

[108] Tanaka KK, Hall JK, Troy AA, Cornelison DDW, Majka SM, Olwin BB (2009). Syndecan-4-expressing muscle progenitor cells in the SP engraft as satellite cells during muscle regeneration. *Cell Stem Cell*.

[109] Day K, Shefer G, Richardson JB, Enikolopov G, Yablonka-Reuveni Z (2007). Nestin-GFP reporter expression defines the quiescent state of skeletal muscle satellite cells. *Developmental Biology*.

[110] Rossi CA, Pozzobon M, Ditadi A (2010). Clonal characterization of rat muscle satellite cells: proliferation, metabolism and differentiation define an intrinsic heterogeneity. *PLoS ONE*.

[111] Kuang S, Kuroda K, le Grand F, Rudnicki MA (2007). Asymmetric self-renewal and commitment of satellite stem cells in muscle. *Cell*.

[112] Qu-Petersen Z, Deasy B, Jankowski R (2002). Identification of a novel population of muscle stem cells in mice: potential for muscle regeneration. *Journal of Cell Biology*.

[113] Danieli-Betto D, Peron S, Germinario E (2010). Sphingosine 1-phosphate signaling is involved in skeletal muscle regeneration. *American Journal of Physiology—Cell Physiology*.

[114] Ferrari G, Cusella-De Angelis G, Coletta M (1998). Muscle regeneration by bone marrow-derived myogenic progenitors. *Science*.

[115] Palumbo R, Sampaolesi M, de Marchis F (2004). Extracellular HMGB1, a signal of tissue damage, induces mesoangioblast migration and proliferation. *Journal of Cell Biology*.

[116] Bosurgi L, Corna G, Vezzoli M (2012). Transplanted mesoangioblasts require macrophage IL-10 for survival in a mouse model of muscle injury. *The Journal of Immunology*.

[117] Kawada H, Ogawa M (2001). Bone marrow origin of hematopoietic progenitors and stem cells in murine muscle. *Blood*.

[118] Tamaki T, Uchiyama Y, Okada Y (2005). Functional recovery of damaged skeletal muscle through synchronized vasculogenesis, myogenesis, and neurogenesis by muscle-derived stem cells. *Circulation*.

[119] Tilki D, Hohn H, Ergün B, Rafii S, Ergün S (2009). Emerging biology of vascular wall progenitor cells in health and disease. *Trends in Molecular Medicine*.

[120] Campagnolo P, Cesselli D, Al Haj Zen A (2010). Human adult vena saphena contains perivascular progenitor cells endowed with clonogenic and proangiogenic potential. *Circulation*.

[121] Farrington-Rock C, Crofts NJ, Doherty MJ, Ashton BA, Griffin-Jones C, Canfield AE (2004). Chondrogenic and adipogenic potential of microvascular pericytes. *Circulation*.

[122] Paul G, Özen I, Christophersen NS (2012). The adult human brain harbors multipotent perivascular mesenchymal stem cells. *PLoS ONE*.

[123] Conboy IH, Conboy MJ, Smythe GM, Rando TA (2003). Notch-mediated restoration of regenerative potential to aged muscle. *Science*.

[124] Chazaud B, Sonnet C, Lafuste P (2003). Satellite cells attract monocytes and use macrophages as a support to escape apoptosis and enhance muscle growth. *Journal of Cell Biology*.

[125] Mounier R, Chrétien F, Chazaud B (2011). Blood vessels and the satellite cell niche. *Current Topics in Developmental Biology*.

[126] Pacilli A, Pasquinelli G (2009). Vascular wall resident progenitor cells. A review. *Experimental Cell Research*.

[127] Passman JN, Dong XR, Wu S (2008). A sonic hedgehog signaling domain in the arterial adventitia supports resident Sca1+ smooth muscle progenitor cells. *Proceedings of the National Academy of Sciences of the United States of America*.

[128] Tidball JG (2005). Inflammatory processes in muscle injury and repair. *American Journal of Physiology—Regulatory Integrative and Comparative Physiology*.

[129] Arnold L, Henry A, Poron F (2007). Inflammatory monocytes recruited after skeletal muscle injury switch into antiinflammatory macrophages to support myogenesis. *Journal of Experimental Medicine*.

[130] Villalta SA, Nguyen HX, Deng B, Gotoh T, Tidbal JG (2009). Shifts in macrophage phenotypes and macrophage competition for arginine metabolism affect the severity of muscle pathology in muscular dystrophy. *Human Molecular Genetics*.

[131] Bosurgi L, Manfredi AA, Rovere-Querini P (2011). Macrophages in injured skeletal muscle: a perpetuum mobile causing and limiting fibrosis, prompting or restricting resolution and regeneration. *Frontiers in Immunology*.

[132] Warren GL, Hulderman T, Jensen N (2002). Physiological role of tumor necrosis factor alpha in traumatic muscle injury. *The FASEB Journal*.

[133] Chen S, Gerken E, Zhang Y (2005). Role of TNF-*α* signaling in regeneration of cardiotoxin-injured muscle. *American Journal of Physiology—Cell Physiology*.

[134] Langen RCJ, van der Velden JLJ, Schols AMWJ, Kelders MCJM, Wouters EFM, Janssen-Heininger YMW (2004). Tumor necrosis factor-alpha inhibits myogenic differentiation through MyoD protein destabilization. *The FASEB Journal*.

[135] Chen S, Jin B, Li Y (2007). TNF-*α* regulates myogenesis and muscle regeneration by activating p38 MAPK. *American Journal of Physiology—Cell Physiology*.

[136] Baeza-Raja B, Muñoz-Cánoves P (2004). p38 MAPK-induced nuclear factor-kappaB activity is required for skeletal muscle differentiation: role of interleukin-6. *Molecular Biology of the Cell*.

[137] Cheng M, Nguyen M, Fantuzzi G, Koh TJ (2008). Endogenous interferon-*γ* is required for efficient skeletal muscle regeneration. *American Journal of Physiology—Cell Physiology*.

[138] Ma J, Wang Q, Fei T, Han JJ, Chen Y (2007). MCP-1 mediates TGF-*β*-induced angiogenesis by stimulating vascular smooth muscle cell migration. *Blood*.

[139] Shireman PK, Contreras-Shannon V, Ochoa O, Karia BP, Michalek JE, McManus LM (2007). MCP-1 deficiency causes altered inflammation with impaired skeletal muscle regeneration. *Journal of Leukocyte Biology*.

[140] Hong KH, Ryu J, Han KH (2005). Monocyte chemoattractant protein-1-induced angiogenesis is mediated by vascular endothelial growth factor-A. *Blood*.

[141] Pelosi L, Giacinti C, Nardis C (2007). Local expression of IGF-1 accelerates muscle regeneration by rapidly modulating inflammatory cytokines and chemokines. *The FASEB Journal*.

[142] Novak ML, Bryer SC, Cheng M (2011). Macrophage-specific expression of urokinase-type plasminogen activator promotes skeletal muscle regeneration. *The Journal of Immunology*.

[143] Sisson TH, Nguyen M, Yu B, Novak ML, Simon RH, Koh TJ (2009). Urokinase-type plasminogen activator increases hepatocyte growth factor activity required for skeletal muscle regeneration. *Blood*.

[144] Rodeheffer MS (2010). Tipping the scale: muscle versus fat. *Nature Cell Biology*.

[145] Moyer AL, Wagner KR (2011). Regeneration versus fibrosis in skeletal muscle. *Current Opinion in Rheumatology*.

[146] Czerwinska AM, Streminska W, Ciemerych MA, Grabowska I (2012). Mouse gastrocnemius muscle regeneration after mechanical or cardiotoxin injury. *Folia Histochemica et Cytobiologica*.

[147] Zhou Z, Cornelius CP, Eichner M, Bornemann A (2006). Reinnervation-induced alterations in rat skeletal muscle. *Neurobiology of Disease*.

[148] Hiatt K, Lewis D, Shew M, Bijangi-Vishehsaraei K, Halum S (2012). Ciliary neurotrophic factor (CNTF) promotes skeletal muscle progenitor cell (MPC) viability via the phosphatidylinositol 3-kinase-Akt pathway. *Journal of Tissue Engineering and Regenerative Medicine*.

[149] Lee N, Spearry RP, Leahy KM (2013). Muscle ciliary neurotrophic factor receptor alpha promotes axonal regeneration and functional recovery following peripheral nerve lesion. *Journal of Comparative Neurology*.

[150] Menetrey J, Kasemkijwattana C, Day CS (2000). Growth factors improve muscle healing in vivo. *Journal of Bone and Joint Surgery B*.

[151] Heumann R, Korsching S, Bandtlow C, Thoenen H (1987). Changes of nerve growth factor synthesis in nonneuronal cells in response to sciatic nerve transection. *Journal of Cell Biology*.

[152] Lavasani M, Lu A, Peng H, Cummins J, Huard J (2006). Nerve growth factor improves the muscle regeneration capacity of muscle stem cells in dystrophic muscle. *Human Gene Therapy*.

[153] Li Y, Thompson WJ (2011). Nerve terminal growth remodels neuromuscular synapses in mice following regeneration of the postsynaptic muscle fiber. *The Journal of Neuroscience*.

